# Dataset for corporate valuation and analyses of peer effects in corporate practices and local factors favoring innovation

**DOI:** 10.1016/j.dib.2016.12.007

**Published:** 2016-12-10

**Authors:** Andrea Carosi

**Affiliations:** University of Sassari, Department of Economics and Business, 25 Via Muroni, 07100 Sassari, Italy

**Keywords:** Corporate market value, Local valuation, Geographical regression, Peer effect

## Abstract

This data article provides cross-sectionals on the local values of the coefficients of ROE, R&D-TO-SALES, and TOTAL ASSET as regressors of the MARKET-TO-BOOK ratio and is related to the research article entitled “Do Local Causations Matter? The Effect of Firm Location on the Relations of ROE, R&D, and Firm Size with Market-to-Book” (A. Carosi, 2016) [1]. The data are aggregated at the regional level (NUTS2). The reported data are the regional average values of the coefficients of ROE, R&D-TO-SALES, and LN(TOTAL ASSET) on LN(MARKET-TO-BOOK), estimated upon the Italian non-financial listed firms in 1999–2007. Local coefficient estimates for family firms and utilities are also provided.

**Specifications Table**

**Table:** 

Subject area	*Economics*
More specific subject area	*Corporate finance, corporate valuation, R&D*
Type of data	*Tables*
How data was acquired	*Data was acquired by merging the Italian regulator׳s (Consob) database, Osiris (Bureau Van Dijk), and company annual reports, for data on headquarters locations; the electronic archive of IlSole24Ore, for press coverage; and Datastream and Worldscope (Thompson Financial), for all accounting and financial information. Google Maps provided the geographic coordinates (i.e. latitude and longitude) of each sampled firm headquarters.*
Data format	*Aggregated, processed*
Experimental factors	*The sample was extracted by merging information from Consob database, Osiris, company annual reports, IlSole24Ore, and Datastream and Worldscope. Sample construction involved various consistent checks. The final dataset made available is a long panel at the firm level from 1999 to 2007.*
Experimental features	*This is the first dataset reporting regression coefficients estimated at the firm location level.*
Data source location	*Italy*
Data accessibility	*Data are available within this article*

**Value of the data**

• First of this kind, the data are the regional (NUTS2) average values of the sensitivity of the MARKET-TO-BOOK to factors very commonly used in corporate valuation, such as ROE (for firm profitability), R&D-TO-SALES (for firm growth opportunities and innovation), and the TOTAL ASSET (for firm size).
• The sensitivities are calculated upon the population of the listed Italian manufacturing firms (1999–2007), but can also be applied to non-financial private firms; in addition, specific local sensitivities for family firms and utilities are further provided.
• In light of the differences in the local sensitivities across different regions, valuation practices are expected to make reference and widely use these data.
• Local sensitivities account for usually unobservable and empirically difficult to address local factors, such as peer effects and factors favoring innovation. As such, these data offer the optimal empirical base for further research in these fields.

The sensitivities are calculated upon the population of listed Italian manufacturing firms (1999–2007), but can also be applied to non-financial private firms; in addition, specific local sensitivities for family firms and utilities are provided.

## 1 Data

The data on the coefficients on ROE, R&D-TO-SALES, and LN(TOTAL ASSET) with the LN(MARKET-TO-BOOK) are available at region-level (NUTS2). The reported data are estimated upon the Italian non-financial listed firms (1999–2007); nevertheless, this data can also be applied to private firms. Furthermore, the average regional coefficients on ROE, R&D-TO-SALES, and TOTAL ASSET for manufacturing firms (Table 1), estimates of the average regional values of coefficients for family firms (Table 2) and utilities (Table 3) are available. In tables, standard deviations of the regional coefficients are reported, and regions with no listed firms are evaluated according to the domestic average values (in italics).

## 2 Experimental design, materials and methods

The data are estimated upon the sample of 1652 firm-year observations on non-financial firms issuing common stocks on the Milan Stock Exchange (MSE) from 1999 to 2007, with ROE between plus and minus one, and headquartered in Italy. Specific data for the subsamples of family firms and utilities are also estimated and provided. Financial firms are SIC 6000–6999. Family firms are firms where the ultimate owner is a family or in which family members hold positions on the board of directors [2]. Utility firms are SIC 4000–4999.

The data are estimated using a Geographically Weighted Regression (GWR) model with adaptive Gaussian kernels according to CV bandwidth selection method [3], and latitude and longitude of sampled firm headquarters locations. The logarithmic transformation of the market-to-book ratio (LN(MARKET-TO-BOOK)) is the dependent variable; the key explanatory variables are equity profitability (ROE), firm future growth opportunities (R&D-TO-SALES), and firm size (LN(TOTAL ASSET)); control variables include firm age [4], as the natural log of firm years since firm foundation, firm press coverage [5], as the natural log of the number of Sole24Ore (the most prominent Italian financial newspaper) articles that mention the firm name during the previous year, a dummy variable which equals one if the company does not report R&D [6], four-digit SIC industry dummies, exchange segment listing dummies, and year dummies. In the GWR model, coefficients on ROE, R&D-TO-SALES, and LN(SIZE) are space-variant, while others explanatory variables are assumed space-invariant. GWR estimates parameters at a point in space since observations close to this point have a greater weight in the estimation than the farthest ones. Ultimately, with respect to each space-variant relation, GWR provides a set of local parameter estimates, one value for each location.

## Figures and Tables

**Table 1:** Average regional coefficients of ROE, R&D-TO-SALES, and TOTAL ASSET on MARKET-TO-BOOK for manufacturing firms.

**Region (Italy - NUTS2)**	**No of Observation**	**LN(MARKET-TO-BOOK)**					
		**Coefficient on ROE**		**Coefficient on R&D-TO-SALES**		**Coefficient on LN(TOTAL ASSET)**	
		***μ***	***σ***	***μ***	***σ***	***μ***	***σ***
Abruzzo	2	0.540	0.0	−1.112	0.0	−0.147	0.0
*Aosta Valley*	*0*	*0.503*	*0.170*	−*0.015*	*1.829*	−*0.145*	*0.003*
Apulia	2	0.397	0.0	−0.448	0.0	−0.144	0.0
*Basilicata*	*0*	*0.503*	*0.170*	−*0.015*	*1.829*	−*0.145*	*0.003*
*Calabria*	*0*	*0.503*	*0.170*	−*0.015*	*1.829*	−*0.145*	*0.003*
Campania	16	0.784	0.011	4.135	0.097	−0.139	0.0
Emilia-Romagna	228	0.709	0.121	3.333	1.091	−0.140	0.001
Friuli-Venezia Giulia	42	0.493	0.041	−0.804	0.191	−0.146	0.0
Lazio	222	0.511	0.040	−1.379	0.099	−0.148	0.001
Liguria	34	0.453	0.001	1.344	0.063	−0.141	0.0
Lombardy	620	0.356	0.121	−1.090	0.789	−0.147	0.002
Marche	32	0.731	0.162	1.841	2.154	−0.143	0.003
Molise	10	0.535	0.016	0.608	0.299	−0.143	0.0
Piedmont	207	0.664	0.100	0.381	0.991	−0.145	0.001
Sardinia	11	0.496	0.016	0.040	0.164	−0.145	0.0
Sicily	6	0.408	0.0	−0.890	0.0	−0.146	0.0
Trentino Alto Adige	3	0.544	0.0	−1.056	0.0	−0.146	0.0
Tuscany	89	0.450	0.012	1.204	0.691	−0.141	0.001
*Umbria*	*0*	*0.503*	*0.170*	−*0.015*	*1.829*	−*0.145*	*0.003*
Veneto	128	0.543	0.035	−0.941	0.469	−0.147	0.001
*Regional Min*		*0.356*		−*1.379*		−*0.148*	
*Regional Max*		*0.784*		*4.135*		−*0.139*	

**Table 2:** Average regional coefficients of ROE, R&D-TO-SALES, and TOTAL ASSET on MARKET-TO-BOOK for family firms.

**Region (Italy - NUTS2)**	**No of Observation**	**LN(MARKET-TO-BOOK)**					
		**Coefficient on ROE**		**Coefficient on R&D-TO-SALES**		**Coefficient on LN(TOTAL ASSET)**	
		***μ***	***σ***	***μ***	***σ***	***μ***	***σ***
*Abruzzo*	*0*	*0.077*	*0.032*	*0.049*	*0.052*	−*0.207*	*0.068*
*Aosta Valley*	*0*	*0.077*	*0.032*	*0.049*	*0.052*	−*0.207*	*0.068*
Apulia	2	0.102	0.0	0.068	0.0	−0.201	0.0
*Basilicata*	*0*	*0.077*	*0.032*	*0.049*	*0.052*	−*0.207*	*0.068*
*Calabria*	*0*	*0.077*	*0.032*	*0.049*	*0.052*	−*0.207*	*0.068*
Campania	9	0.102	0.0	0.094	0.0	−0.171	0.0
Emilia-Romagna	191	0.069	0.032	0.090	0.018	−0.116	0.034
Friuli-Venezia Giulia	8	0.120	0.005	0.060	0.002	−0.215	0.0
Lazio	127	0.095	0.002	0.104	0.001	−0.167	0.000
Liguria	9	0.072	0.0	0.027	0.0	−0.259	0.0
Lombardy	395	0.058	0.023	0.002	0.045	−0.264	0.047
Marche	26	0.135	0.002	0.089	0.004	−0.167	0.004
Molise	1	0.112	0.0	0.096	0.0	−0.164	0.0
Piedmont	108	0.106	0.004	0.019	0.002	−0.265	0.004
Sardinia	11	0.077	0.000	0.053	0.000	−0.228	0.0
Sicily	4	0.081	0.0	0.072	0.0	−0.205	0.0
Trentino Alto Adige	3	0.084	0.0	0.059	0.0	−0.208	0.0
Tuscany	58	0.042	0.015	0.109	0.012	−0.142	0.015
*Umbria*	*0*	*0.077*	*0.032*	*0.049*	*0.052*	−*0.207*	*0.068*
Veneto	107	0.112	0.022	0.068	0.005	−0.190	0.017
*Regional Min*		*0.042*		*0.002*		−*0.265*	
*Regional Max*		*0.135*		*0.104*		−*0.116*	

**Table 3:** Average regional coefficients of ROE, R&D-TO-SALES, and TOTAL ASSET on MARKET-TO-BOOK for utilities.

**Region (Italy - NUTS2)**	**No of Observation**	**LN(MARKET-TO-BOOK)**					
		**Coefficient on ROE**		**Coefficient on R&D-TO-SALES**		**Coefficient on LN(TOTAL ASSET)**	
		***μ***	***σ***	***μ***	***σ***	***μ***	***σ***
*Abruzzo*	*0*	*0.186*	*0.001*	*0.039*	*0.001*	−*0.244*	*0.004*
*Aosta Valley*	*0*	*0.186*	*0.001*	*0.039*	*0.001*	−*0.244*	*0.004*
*Apulia*	*0*	*0.186*	*0.001*	*0.039*	*0.001*	−*0.244*	*0.004*
*Basilicata*	*0*	*0.186*	*0.001*	*0.039*	*0.001*	−*0.244*	*0.004*
*Calabria*	*0*	*0.186*	*0.001*	*0.039*	*0.001*	−*0.244*	*0.004*
*Campania*	*0*	*0.186*	*0.001*	*0.039*	*0.001*	−*0.244*	*0.004*
Emilia-Romagna	8	0.184	0.001	0.038	0.000	−0.241	0.002
Friuli-Venezia Giulia	7	0.185	0.0	0.038	0.0	−0.236	0.0
Lazio	26	0.188	0.000	0.037	0.000	−0.238	0.000
Liguria	23	0.186	0.000	0.040	0.0	−0.248	0.0
Lombardy	64	0.185	0.000	0.040	0.000	−0.246	0.001
*Marche*	*0*	*0.186*	*0.001*	*0.039*	*0.001*	−*0.244*	*0.004*
*Molise*	*0*	*0.186*	*0.001*	*0.039*	*0.001*	−*0.244*	*0.004*
Piedmont	20	0.186	0.0	0.040	0.0	−0.248	0.0
*Sardinia*	*0*	*0.186*	*0.001*	*0.039*	*0.001*	−*0.244*	*0.004*
*Sicily*	*0*	*0.186*	*0.001*	*0.039*	*0.001*	−*0.244*	*0.004*
*Trentino Alto Adige*	*0*	*0.186*	*0.001*	*0.039*	*0.001*	−*0.244*	*0.004*
*Tuscany*	*0*	*0.186*	*0.001*	*0.039*	*0.001*	−*0.244*	*0.004*
*Umbria*	*0*	*0.186*	*0.001*	*0.039*	*0.001*	−*0.244*	*0.004*
Veneto	2	0.183	0.0	0.038	0.0	−0.238	0.0
*Regional Min*		*0.183*		*0.037*		−*0.248*	
*Regional Max*		*0.188*		*0.040*		−*0.236*	
