# Supplementary material for: Dataset for corporate valuation and analyses of peer effects in corporate practices and local factors favoring innovation
Source: Data Brief. 2016 Dec 10;10:325–9. doi: 10.1016/j.dib.2016.12.007 (PMC5157703; doi:10.1016/j.dib.2016.12.007)

**AUTHOR DECLARATION**

I wish to confirm that there are no known conflicts of interest associated with this publication and there has been no significant financial support for this work that could have influenced its outcome.

I confirm that the manuscript has been read and approved by all named authors and that there are no other persons who satisfied the criteria for authorship but are not listed.

I confirm that I have given due consideration to the protection of intellectual property associated with this work and that there are no impediments to publication, including the timing of publication, with respect to intellectual property. In so doing I confirm that I have followed the regulations of my institution concerning intellectual property.

I understand that the Corresponding Author is the sole contact for the Editorial process (including Editorial Manager and direct communications with the office). He/she is responsible for communicating with the other authors about progress, submissions of revisions and final approval of proofs.

Signed by all authors as follows:

November 29, 2016

Andrea Carosi


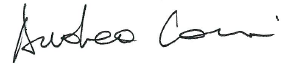

Supplement: Supplementary file 1 — Supplementary material [file mmc1.docx]
